# Genetic structure and conservation implications of *Lancea tibetica* (Mazaceae), a traditional Tibetan medicinal plant endemic to the Qinghai- Tibet Plateau

**DOI:** 10.1186/s12870-025-06258-7

**Published:** 2025-02-18

**Authors:** Mingze Xia, Xiaofeng Chi, Jingya Yu, Yun Han, Shuang Han, Shilong Chen, Yan Li, Faqi Zhang

**Affiliations:** 1 School of Pharmacy & College of Traditional Chinese Medicine, Shandong Second Medical University, Weifang, 261053 China; 2 Key Laboratory of Adaptation and Evolution of Plateau Biota, Northwest Institute of Plateau Biology & Institute of Sanjiangyuan National Park, Chinese Academy of Sciences, Xining, 810008 China; 3 University of Chinese Academy of Sciences, Beijing, 100039 China; 4 Qinghai Provincial Key Laboratory of Crop Molecular Breeding, Xining, 810008 China

**Keywords:** *Lancea tibetica*, Allopatric divergence, RAD-seq, Genetic structure, Qinghai-Tibet Plateau, Species distribution modeling

## Abstract

**Background:**

Allopatric divergence is often initiated by geological uplift and climate oscillations. Qinghai-Tibet Plateau is an excellent place for such research because the plants of this area have experienced such historical processes as glacial contraction, interglacial expansion and geographical isolation. Here in this study, we used Genotyping-By-Sequencing data to investigate allopatric divergence of *Lancea tibetica*, an endemic herb to the Qinghai-Tibet Plateau. A total of 12,005 high-quality single nucleotide polymorphisms were obtained from 183 individuals of 23 natural distribution areas.

**Results:**

Our results confirm that *L. tibetica* is divided into Northern and Southern groups, separated by the Tangula Mountains, Nyainqentanglha Mountains, and the Salween River. Demographic modeling indicated a bottleneck event around 300 kya, followed by gene flow and a recent expansion in both groups. Geographic isolation and climatic variation are likely the primary factors shaping the population structure of this species. Species distribution models reveal that elevation is the most significant factor influencing the distribution of *L. tibetica*, followed by precipitation and temperature. In scenarios of future global warming, suitable habitats for *L. tibetica* are likely to be significantly reduced, with an anticipated migration to higher altitudes. Given the current and projected distribution patterns of *L. tibetica*, the implementation of in-situ conservation and commercial cultivation measures is particularly urgent.

**Conclusions:**

Our study contributes insights into understanding the genetic variation and distribution pattern of species in the Qinghai-Tibet Plateau and its adjacent areas, serving as a valuable reference for future conservation efforts.

**Supplementary Information:**

The online version contains supplementary material available at 10.1186/s12870-025-06258-7.

## Background

The interplay between geographic and environmental factors can significantly impact the genetic structure and evolutionary history of a species [1]. Therefore, regions that have experienced complex orogeny and fluctuating climates offer an ideal platform for investigating fundamental questions in evolutionary biology, such as adaptive evolution, species diversity, and the effects of geoclimatic changes on biological evolution [2, 3]. The Qinghai-Tibet Plateau (QTP) is a renowned global hotspot for alpine plant biodiversity, characterized by a high degree of biogenic complexity and species richness [4, 5]. In recent decades, numerous biogeographical studies have been conducted in the QTP region, aimed at exploring the connection between complex orogeny, associated climate fluctuations, and species diversity [6–13]. Multiple theoretical models suggest that the formation process of plant diversity in the QTP has been long-term and complex. For example, the Flickering‐ Connectivity model indicates that climatic oscillations during the Pleistocene led to geographic isolation and connectivity among alpine taxa, promoting species diversity [14]. The Mountain Biodiversity model proposes that the steep ecological gradients, rapid climate oscillations, and temporal evolution of high-altitude terrains collectively contributed to the diversification of plant assemblages on the QTP [15]. However, it is important to note that species diversity in the region is shaped by a long and complex history, and different species may have distinct origins and patterns of diversification [16, 17]. Furthermore, most studies to date have focused on the historical changes in species distribution patterns, rather than the future distribution patterns and threatened status of species under the influence of global climate change.

In the past few decades, the cost of obtaining sufficient data to conduct population genetics studies has been prohibitive. However, with the development of high-throughput next generation sequencing (NGS), it is now possible to study species diversity using a large number of molecular markers. One such technology, genotyping-by-sequencing (GBS, can be also called RAD-seq), has been widely utilized for the analysis of complex genetic structures in populations [18–24]. GBS enables the generation of a significant number of single nucleotide polymorphism (SNP) markers from species without reference genomes, which are suitable for applications such as genetic mapping, phylogenetic analysis, and diversity analysis [25]. In this study, we utilized GBS data to evaluate the genetic structure and evolutionary history of *Lancea tibetica* in the QTP. This species is considered a valuable model for the study of allopatric divergence, as it is an ancient species on the QTP and has experienced the impact of various geoclimatic events such as uplift of the crust and climatic fluctuations during glacial and interglacial periods [26].

*Lancea tibetica* Hook. f. et Thoms is in the genus *Lancea*, which is a small genus that only has two species (*L. tibetica* and *L. hirsuta* Bonati) [27]. As a traditional Tibetan medicinal plant, *L. tibetica* has been used in the treatment of leukemia, intestinal angina, heart disease, and cough for a long time [28]. Thus, local residents have been harvesting the plant for medicinal use, which has adversely affected the survival of *L. tibetica*. According to herbarium records and our field observations, the distribution of *L. tibetica* extends from the Qilian Mountains in northwestern China to the northern foothills of the Himalayan Mountains and reaches the eastern margin of the QTP. This distribution encompasses a diverse range of elevations, ranging from 2000 to 4500 m [27]. Hence, the species spans two significant biodiversity hotspots—the Himalayan region and the Hengduan Mountains—encompassing the majority of habitats in the QTP. The geographical distribution of *L. tibetica* covers the predominant habitats in the QTP region, rendering it an excellent material for studying speciation and adaptive differentiation in this area. Previous studies have used gene fragment markers to investigate population differentiation in *L. tibetica* [26]. The results indicate that around 8.63 million years ago, the Northern and Southern populations of *L. tibetica* began to diverge, suggesting that geographic isolation played a role in promoting the diversification and evolution of the species [26]. However, due to the limited number of genetic markers, previous studies were unable to comprehensively analyze the dynamic history of *L. tibetica* populations. Therefore, this study aims to conduct an in-depth analysis of the population structure of *L. tibetica* using a large number of molecular markers to explore the historical dynamics of the populations. Additionally, although this species is widely used for medicinal purposes, no conservation studies have been conducted to date. This research intends to employ ecological niche modeling to predict future changes in the population distribution of *L. tibetica* and to provide corresponding conservation recommendations.

The present study aims to further confirm the driving factors behind the genetic differentiation patterns of *L. tibetica* in the QTP region. To achieve this goal, we obtained and analyzed SNP data from 183 individuals, which were sampled across almost the entire geographical distribution of the species. The objective of our analysis was to investigate the influence of environmental and climatic factors on the distribution area of *L. tibetica* and to simulate the potential distribution of the species under future climate conditions. This study seeks to address the following questions: (1) What are the primary factors influencing the historical dynamics and distribution patterns of *L. tibetica* populations within the context of the complex geological history and climatic conditions of the QTP? (2) Under projected future climate scenarios, will the distribution of this medicinal plant face potential threats? If so, what specific conservation strategies should be implemented?

## Methods

### Sample collection, DNA extraction

**Fig. 1:**
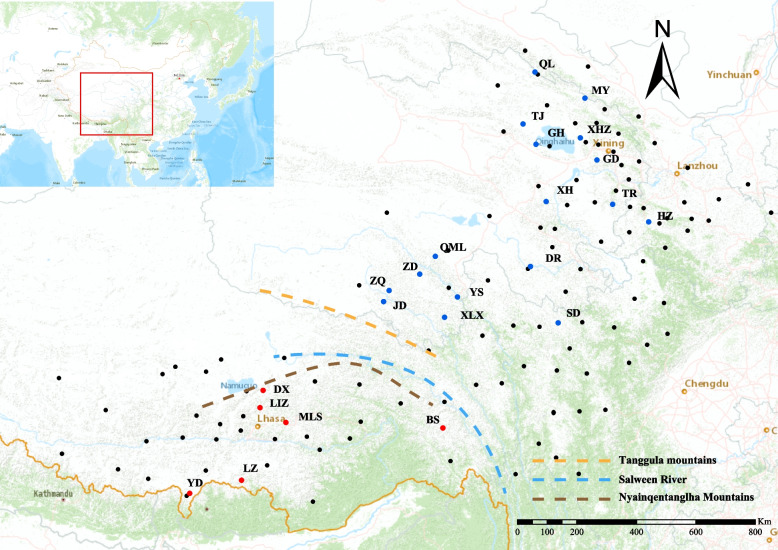
Sampling localities of *Lancea tibetica* across entire distribution range, with populations colored to distinguish the Southern group (red), Northern group (blue) and herbarium records in the Chinese Virtual Herbarium (black)

A total of 183 individuals were sampled from 23 natural distribution areas. Sampling locations were chosen based on existing occurrence records of the Flora of China [27] and herbarium records from the Chinese Virtual Herbarium, covering the major distribution range of *L. tibetica* (Fig. 1). Fresh leaves were collected, dried in silica gel and kept at −20℃ until DNA extraction. All the sampling locations were geo-referenced, and voucher specimens were deposited into the Qinghai-Tibet Plateau Museum of Biology (HNWP), Northwest Institute of Plateau Biology, Chinese Academy of Sciences (Table 1). These samples were identified by Professor Faqi Zhang at Northwest Institute of Plateau Biology, Chinese Academy of Sciences. Genomic DNA was extracted from approximately 20 mg of dried leaf using a modified cetyltrimethylammonium bromide (CTAB) method [29]. The extracted DNA was quantified, and its quality was evaluated with agarose gel electrophoresis, Nanodrop (Wilmington, DE, USA), and Qubit 2.0 (Life Technologies, CA, USA). High-quality DNA samples were then applied to prepare GBS libraries.

**Table 1:** List of the sampling sites, population code, voucher no., and number of samples analyzed of *Lancea tibetica*

P	Location	Voucher No	Latitude (N)	Longitude (E)	Elevation (m)	N
XH	Xinghai, QH	Zhang2014089	35°20′	99°55′	3622	8
TR	Tongren, QH	Zhang2014151	35°16′	101°55′	3036	8
TJ	Tianjun, QH	Zhang2014262	37°12′	99°13′	3340	8
MY	Menyuan, QH	Zhang2014341	37°51′	101°05′	3636	8
XLX	Xialaxiu, QH	Chen2014637	32°23′	96°48′	3770	8
QML	Qumalai, QH	Chen2014684	33°58′	96°34′	4570	8
DR	Dari, QH	Zhang2015107	33°41′	99°26′	4028	8
GD	Guide, QH	Zhang2014189	36°21′	101°26′	3782	8
GH	Gonghe, QH	Zhang2014378	36°46′	99°40′	3396	8
JD	Jieduo, QH	Zhang2016753	32°52′	95°00′	4327	8
QL	Qilian, QH	Zhang2014317	38°26′	99°33′	3296	8
YS	Yushu, QH	Chen2014591	32°55′	97°13′	3667	8
ZD	Zhiduo, QH	Chen2014667	33°29′	96°05′	4370	8
ZQ	Zhaqing, QH	Zhang2016722	33°5′	95°9′	4289	8
XHZ	Xihaizhen, QH	Zhang2014276	36°52′	100°54′	3137	8
HZ	Hezuo, GS	Fu2016012	34°50′	103°00′	3220	8
SD	Seda, SC	Fu2016040	32°17′	100°16′	3926	8
LZ	Luozha, XZ	Chen2013394	28°08′	90°41′	4566	8
MLS	Milashan, XZ	Zhang2014019	29°42′	92°03′	4136	8
BS	Basu, XZ	Chen2014298	29°31′	96°46′	4140	8
YD	Yadong, XZ	Chen2014498	27°47′	89°08′	4350	7
LIZ	Linzhou, XZ	Fu2016208	30°04′	91°16′	4232	8
DX	Dangxiong, XZ	Fu2016210	30°32′	91°20′	4381	8

*P.* Population code, *N* number of samples, *QH* Qinghai Province, China, *GS* Gansu Province, China, *SC* Sichuan Province, China, *XZ* Tibet, China

### Library preparation, sequencing and SNP calling

For GBS library construction and sequencing, genomic DNA from individual samples was digested with the *MseI* restriction enzyme. The digested DNA fragments were ligated to the Illumina sequencing adapters and sample-specific barcodes with T4 DNA ligase (New England Biolabs, NEB) [18]. Recycled and purified the 350–400 bp adapter-ligated DNA fragments by agarose gel electrophoresis. The purified DNA fragments were amplified and subjected to paired-end 150 bp sequencing on the NovaSeq 6000 (Illumina Inc., San Diego, CA, USA) sequencing platform.

Raw sequencing data were converted from original image data (BCL files) to FASTQ format using bcl2fastq v. 2.20 software, with parameters set according to the guidelines provided by Illumina (https://support.illumina.com/downloads/bcl2fastq-conversion-software-v2-20.html). The data were then filtered to remove adapters and low-quality reads using Trimmomatic v. 0.33 [30] with the following parameters: reads containing adapter sequences were removed; reads with more than 10% of their length composed of 'N' bases were discarded; paired reads were removed if one of the reads had more than 50% of its length consisting of low-quality bases (≤ Q5). We chose assembled results of sample XH7 as the reference. The remaining samples were mapped to the reference genome using BWA v0.7.8 [31] (parameter: mem -t 4 -k 32 -M -R) and this mapping result was analyzed to evaluate the sequencing condition. The sample mapping rate ranged from 73.70% to 92.36%, the coverage more than 52.81% at 1X depth and 35.87% at 4X depth. We used SAMtools v1.3.1 [32] to discover SNPs on a population scale, and then detect the polymorphic site by Bayesian model. The “mpileup” command was used to identify SNPs (parameter: -m 2—F 0.002—d 1000). To exclude SNP calling errors caused by incorrect mapping of repeat regions, we set the filter rules (coverage depth ≥ 2 and ≤ 1000, genotype quality ≥ 5, minor allele frequency > 0.01, miss < 0.6) to ensure the high quality of SNPs for phylogenetic analysis.

### Population structure analysis

Population structure was preliminarily assessed by analyzing the most likely number of genetic clusters (K). We used VCFTOOLS version 0.1.15 [33] to convert the high-quality SNPs data into input data of PLINK version 1.90 [34]. After filtering the linked sites (parameter: –indep-pairwise 50 10 0.2), we used ADMIXTURE v.1.23 [35] to estimate population structure of with K‐value setting ranging from 1 to 10. The optimal number of groups was determined according to the cross-validation error (CV error) value. We also utilized SAMOVA version 2.0 [36] to analyze the population structure using default parameters. In addition, population structure was also performed by the principal component analysis (PCA) using GCTA version 1.26.0 [37].

### Phylogenetic tree construction

Phylogenetic tree was constructed to clarify the genetic relationships among samples. TreeBest v. 1.9.2 was employed to obtain a genetic distance matrix through 1,000 bootstrap replicates (http://treesoft.sourceforge.net/treebest.shtml). Subsequently, after converting the file format, the neighbor-joining tree was constructed using MEGA7 under default parameters [38].

### Genetic diversity analysis

We calculated the genetic diversity index of the population, including expected heterozygosity (*He*), observed heterozygosity (*Ho*), nucleotide diversity (π), and inbreeding coefficients (*F*_IS_) with populations module in the Stacks v.2.55 [39]. We conducted the Hardy–Weinberg equilibrium (HWE) using the exact test implemented in the R package “HardyWeinberg” [40]. In addition, a bootstrap approach test to examine if the *F*_IS_ coefficients are significantly different from zero using the boot.ppfis function in the R package “hierfstat” [41]. In order to evaluate the correlation between genetic distance and geographical distance, the genetic distance matrix between 23 populations was calculated by using the software Arlequin V.3.5 [42]. Then, the Mantel test of the correlation between genetic distance and geographical distance was carried out in GenAlEx v.6.5 [43].

Based on the results of population structure and phylogenetic analysis, the populations of *L. tibetica* were grouped. To perform the genetic differentiation within populations, between populations within groups, and between groups, the analysis of molecular variance (AMOVA) was calculated in Arlequin v.3.5 [42] with significance tests of variance based on 10,000 permutations.

### Demographic history analyses

To infer the population differentiation history of *L. tibetica*, we used fastsimcoal version 2.7.0.9 [44] to estimate demographic parameters based on the two-dimensional joint-folded site frequency spectrum (2D-SFS), which was calculated using ANGSD [45], as suggested by Excoffier et al. [44]. Based on the results of the population structure analysis in this study, we defined the groupings of *L. tibetica* populations and established eight isolation and migration models between the groups, each assuming different population size changes and gene flow scenarios. These eight models include: isolation with/without gene flow for descendant populations with stable population sizes; isolation with/without gene flow for descendant populations with immediate expansion; isolation with/without gene flow for descendant populations with expansion at a specific time; isolation with/without gene flow for descendant populations experiencing a bottleneck followed by expansion at a specific time. Each model was executed 60 times, employing 100,000 coalescent simulations and 40 cycles of the conditional maximization algorithm to achieve the global maximum likelihood, as suggested by the manual of fastsimcoal [44]. The best-fit model was selected based on Akaike's Information Criterion (AIC) and Akaike's weight, as suggested by Korneliussen et al. [45].

### Gene flow analysis

Gene flow analysis helps in identifying the degree of genetic connectivity among populations and inferring historical isolation events and migration patterns. To reduce the impact of selection bias, we performed SNP data pruning based on linkage disequilibrium values. The filtering process was performed using the software PLINK version 1.90 with the parameter: –indep-pairwise 50 10 0.1 [34]. Based on the pruned SNP data, we used TreeMix v.1.13 (parameter: -m from 1 to 9) to evaluate gene flow between populations [46]. We used R package “OptM” to estimate the optimal number of migration edges [47]. The direction of gene flow was visualized by R package “plotting_funcs”. MIGRATE v.4.4.3 [48] was utilized to estimate the gene flow between populations, with the following parameters: Number of recorded steps in chain = 10,000; Burn-in for each chain = 100,000; Running multiple replicates = 3.

### Species distribution modeling

In this study, we employed the species distribution modeling (SDM) approach to predict the current (1970–2000 A.D.) and future (2081–2100 A.D.) potential distribution of *L. tibetica*. To this end, we sourced 19 current and future bioclimatic variables from the WorldClim 2.0 database (https://www.worldclim.org/). In order to prevent the occurrence of strong collinearity among environmental variables and to avoid overfitting of the model, we conducted a Pearson correlation analysis on the 19 bioclimatic variables and removed variables with high correlation (Pearson correlation value > 0.7). The final set of bioclimatic variables that were retained for further analysis consisted of 7 variables, including Annual Mean Temperature, Mean Diurnal Range, Annual Precipitation, Precipitation of Driest Month, Precipitation Seasonality, and Precipitation of Coldest Quarter. In addition, we also obtained Potential Evapotranspiration data (https://cgiarcsi.community/) and Digital Elevation Model (DEM, https://download.gebco.net/) data of the study area, and extracted slope and aspect data from DEM.

The potential suitable distribution areas for *L. tibetica* were predicted by importing the adjusted environmental factor data (2.5 arc-min) and species distribution data into MAXENT V. 3.4.4 [49]. Ten cross-validation repetitions were conducted, with all other parameters maintained at their default settings. The contribution of bioclimatic variables to the predictions of distribution patterns was assessed using the jackknife method. The accuracy of the model predictions was evaluated using the area under the curve (AUC) of the receiver operating characteristic (ROC) [50].

To determine the current and future changes in the adaptive habitat of *L. tibetica*, we utilized the ArcGIS V. 10.8.1 software and reclassified the predicted results based on the habitat suitability values. The potential suitable habitats were divided into four categories: high suitability (0.6 < predicted habitat suitability value ≤ 1.0), medium suitability (0.4 < predicted habitat suitability value ≤ 0.6), low suitability (0.2 < predicted habitat suitability value ≤ 0.4), and non-suitable (predicted habitat suitability value < 0.2), were based on related research [51]. Additionally, we calculated the area extent for each of these categories.

## Results

### Sequence assembly and SNP detection

Genotyping by Sequencing was performed to generate sequence data from the 23 *L. tibetica* populations. The initial raw data generated from the GBS sequencing amounted to 180.38 Gb, and after filtering and cleaning, 177.90 Gb of high-quality data were obtained. All the individuals had high quality sequence data (Q20 ≥ 94.47% and Q30 ≥ 85.28%) and a normal distribution of GC content (average from 34% to 36%). After processing the data, an average of 6,758,658.48 clean reads were obtained per individual, totaling 1,236,834,502 clean reads from 1,252,642,566 reads. Finally, 12,005 high-quality SNPs were retained for further analysis, selected from the 214,873 raw SNPs.

### Population structure and phylogenetic analysis

**Fig. 2:**
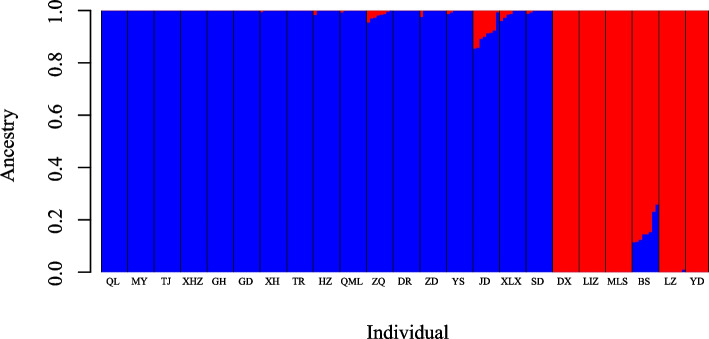
Genetic population groups as determined by ADMIXTURE for K = 2. The length of the different color vertical bars representing the proportion of an ancestor in the individual’s genome. Populations depicted in blue correspond to the Northern group, while those in red represent the Southern group

**Fig. 3:**
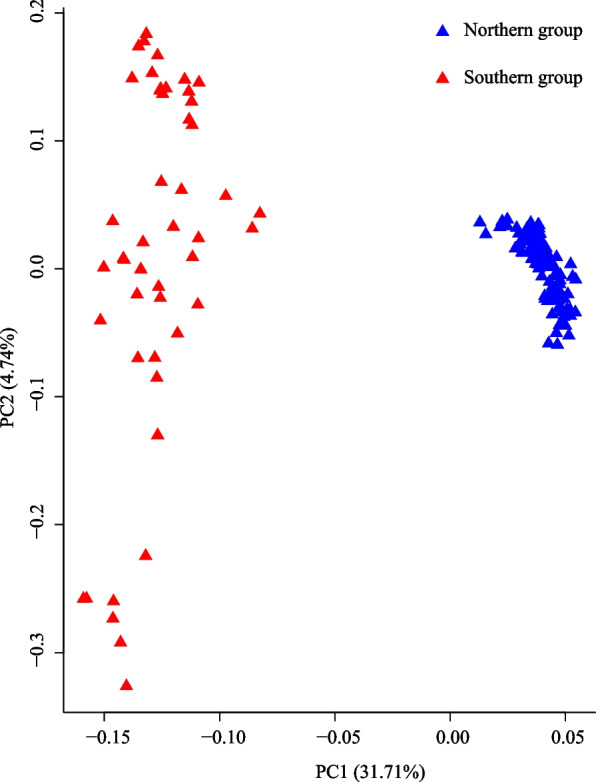
Principal component analyses (PCA) of 23 *Lancea tibetica* populations based on SNP data. The blue and red triangular distributions represent the populations of Northern group and Southern group

The ADMIXTURE method was employed to study the population structure of 23 *L. tibetica* populations. The CV error value indicated that the optimal number of groups was two, with a CV error value of 0.26681 (Additional file 2). The results of ADMIXTURE grouping and SAMOVA analysis confirmed that the *L. tibetica* populations were separated into a Northern group and a Southern group, reflecting their geographical distributions (Figs. 1 and 2, Table 2). The Northern group consisted of populations from Qinghai, Gansu, and Sichuan provinces, while the Southern group included populations from Tibet. This division was also supported by the results of the PCA, which showed a consistent population composition in each group (Fig. 3).

**Table 2:** The genetic diversity values of *Lancea tibetica* populations

Group	Pop ID	π	*He*	*Ho*	*F*_IS_	Percentage of deviating
North	DR	0.12264	0.11373	0.13923	−0.03601	1.55%
	GD	0.11945	0.11091	0.14074	−0.04606	1.68%
	GH	0.11757	0.10918	0.13945	−0.04746	1.23%
	HZ	0.12253	0.11412	0.13845	−0.03438	1.67%
	JD	0.13364	0.12435	0.14891	−0.03178	1.77%
	MY	0.12606	0.11122	0.13976	−0.0285	0.38%
	QL	0.11885	0.11055	0.13585	−0.03651	1.62%
	QML	0.12415	0.10647	0.13497	−0.021	0.26%
	SD	0.12394	0.11432	0.14063	−0.03655	1.41%
	TJ	0.12572	0.1093	0.13289	−0.01514	0.48%
	TR	0.11457	0.09504	0.12515	−0.02153	0.39%
	XH	0.12051	0.10079	0.13013	−0.01991	0.40%
	XHZ	0.11693	0.10883	0.14064	−0.04953	1.96%
	XLX	0.12877	0.10978	0.13588	−0.01374	0.52%
	YS	0.12564	0.11417	0.14375	−0.03789	0.96%
	ZD	0.12388	0.11489	0.14433	−0.0449	2.12%
	ZQ	0.12056	0.11136	0.14521	−0.05238	1.61%
Sourth	BS	0.13844	0.11927	0.14995	−0.02285	0.41%
	DX	0.10509	0.09469	0.13073	−0.05467	1.54%
	LIZ	0.10254	0.09281	0.12556	−0.04914	1.60%
	LZ	0.1141	0.09137	0.12741	−0.02509	0.15%
	MLS	0.11144	0.08999	0.12592	−0.02696	0.33%
	YD	0.11295	0.09182	0.13072	−0.03414	0.28%

Note: π, average nucleotide diversity in this population; *He*, average expected heterozygosity per locus in this population; *Ho*, average expected homozygosity per locus in this population; *F*_IS_, the inbreeding coefficients; Percentage of deviating, the percentage of loci deviating from Hardy–Weinberg equilibrium (HWE)

The neighbor-joining phylogenetic tree showed that individuals within the same population were largely clustered together, with some individuals from populations GD, GH, DR, TR, and TJ clustering with individuals from other populations. The tree separates the individuals into two groups, distinguishing those sampled from Tibet from those collected in other provinces (Additional file 3).

The AMOVA analysis showed that 53.76% of the genetic variation existed within the populations, while 40.86% was between the two groups and 5.38% was within the groups (*p* < 0.001) (Table 3).

**Table 3:** The analysis of molecular variance (AMOVA) for SNP data among Northern and Southern groups

Source of variation	d.f	Sum of squares	Variance component	Percentage of variation (%)	Fixation index
Among groups	1	1341.503	9.37399 Va	40.86	*F*ST = 0.4624* *F*SC = 0.09097* *F*CT = 0.4086*
Within groups	21	671.590	1.23420 Vb	5.38	
Within populations	343	4230.330	12.33332 Vc	53.76	
Total	365	6243.423	22.94152		

Note: *d.f.* degree of freedom. Significance

^*^P < 0.001

### Population genetic diversity analysis

The HWE analysis demonstrates that less than 2.2% of loci per population deviate from HWE (*p* < 0.05). This low deviation rate (consistent across all 23 populations) suggests minimal systematic genotyping errors and aligns with expectations for natural populations experiencing weak evolutionary forces. In addition, the population BS also had the highest average value of *Ho* (0.14995) and the second-highest average value of *He* (0.11927) (only lower than population JD). The average *He* value for the 23 populations was 0.10691, ranging from 0.08999 (MLS) to 0.12435 (JD). The paired t-test revealed a statistically significant difference between *Ho* and *He* across populations (*p* < 0.05). Specifically, the mean *Ho* values were consistently higher than the corresponding *He* values. The *F*_*IS*_ value ranged from −0.05467 (DX) to −0.01374 (XLX). Bootstrap analyses revealed significantly negative *F*_*IS*_ values across all 23 populations. The consistency of these estimates, with all upper confidence limits below zero, indicates systematic heterozygote excess at the population level. The results of the Mantel test indicated a positive correlation between geographical distance and genetic distance (R = 0.59, *P* < 0.01) (Additional file 4).

### Demographic history and gene flow analysis

**Fig. 4:**
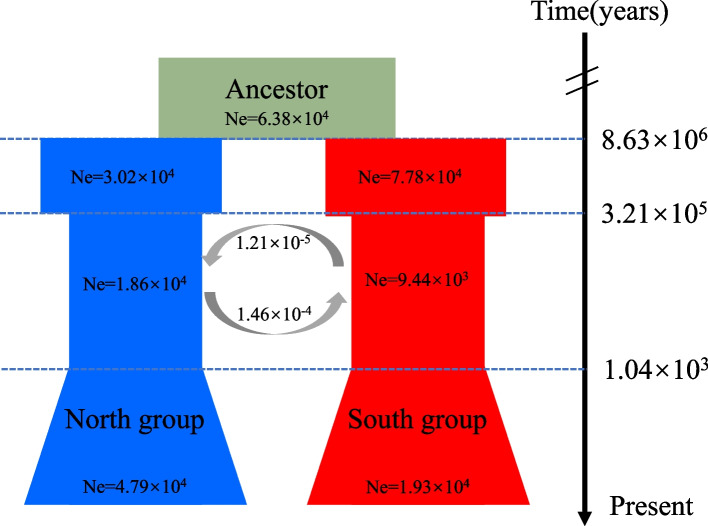
Schematic of the demographic history of *Lancea tibetica*, as depicted by the best model in fastsimcoal 2. Estimated effective population sizes (Ne), divergence time and rates of gene flow (per generation migration rate) are indicated in the schematic

The historical population dynamics of *L. tibetica* were inferred using coalescent simulations in fastsimcoal2. The best model (Akaike's weight = 1; Additional file 1) suggested that, following the separation of the Northern and Southern groups from their ancestral population, there was continuous gene flow between the two groups. Both groups experienced a bottleneck event around 0.32 Ma, resulting in a significant reduction in effective population size. Subsequently, around 1040 years ago, the effective population size began to increase (Fig. 4).

Gene flow analysis using Treemix showed that there were 4 and 2 optimal migration edges in the Northern and Southern groups, respectively (Additional file 5, Additional file 6, Additional file 7, Additional file 8). Additionally, the scaled migration rate (*M*) between populations was calculated using MIGRATE. In the Northern group, gene flow occurred from XLX to SD (*M* = 5.82), from XLX to XH (*M* = 8.59), from XHZ to TJ (*M* = 6.55), and from TR to HZ (*M* = 5.42) (Additional file 5). In the Southern group, gene flow occurred from YD to MLS (*M* = 6.44) and from BS to MLS (*M* = 5.44) (Additional file 6).

### Species distribution models

**Fig. 5:**
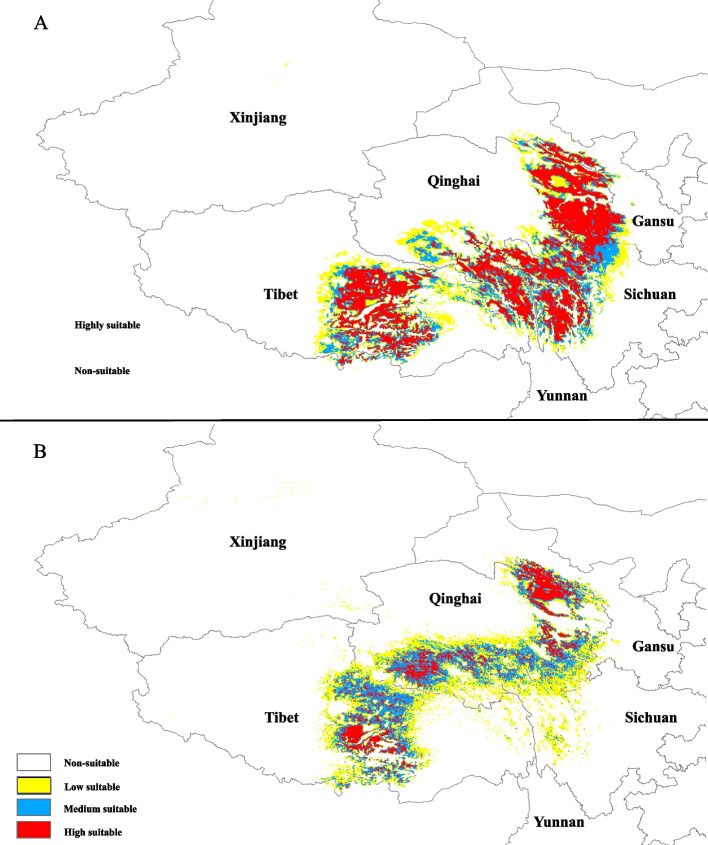
Potential distribution of *Lancea tibetica* under future global warming scenarios. Predicted distributions are shown for (**A**) the present (1970–2000) model, (**B**) the future (2081–2100, SSP2-4.5) model. Grid with warm colors indicates high suitable area and with cold colors indicates low suitable area

The results of the species distribution modeling, using MAXENT, for *L. tibetica* demonstrated an excellent prediction accuracy, with an average AUC value of 0.976 ± 0.009 (as shown in Additional file 9). Variable jackknife analyses indicated that the environmental variables with the highest contribution to the potential distribution model were elevation (36.0%), annual precipitation (20.6%), annual mean temperature (17.8%), and the precipitation of the coldest quarter (10.1%). The potential distribution of *L. tibetica* was simulated under both the current and future climate change scenarios, as depicted in Fig. 5. The results confirmed a significant contraction in the potential distribution in the southern and eastern areas when comparing the future scenario to the present scenario. Under the future climate scenario of SSP2-4.5, the large suitable areas of *L. tibetica* in northwestern Sichuan and eastern Tibet will undergo a substantial reduction. Conversely, some areas previously on the edge of the Tibetan Plateau, such as eastern and southern Qinghai Province, will retreat towards the plateau platform of QTP.

We calculated the different suitability levels of the predicted distribution areas of *L. tibetica* under present and future climate scenarios. The results indicated that the high suitable area currently covers 301,736.11 km^2^, while the medium suitable area is 195,416.66 km^2^ and the low suitable area is 234,149.30 km^2^. However, under the projected future climate change scenario, the high suitable area is reduced to 112,847.22 km^2^, while the medium suitable area increases to 199,513.89 km^2^ and the low suitable area to 290,590.29 km^2^. This results in a decrease of 62.6% in the high suitable area, with a corresponding increase of 24.1% in the low suitable area.

## Discussion

As an endemic species in the QTP and surrounding regions, understanding the genetic distribution patterns of *L. tibetica* can provide valuable insights into the biodiversity of the QTP. The rapid development of high-throughput sequencing technology has enabled researchers to study population genetic diversity and structure based on GBS data. In this study, we sequenced individuals from 23 populations of *L. tibetica* and obtained 214,873 high-quality SNPs for analysis. Our results confirmed a strong correlation between the genetic structure and geographical distribution, leading to the division of *L. tibetica* populations into Northern and Southern groups. Furthermore, our species distribution models indicated that under future scenarios of global climate change, the suitable habitat for *L. tibetica* will significantly shrink and the populations will likely retreat to the plateau regions of the QTP. This study provides new insights into the genetic variation and distribution patterns of species in the QTP and surrounding areas.

### Population genetic structure formation of *L. tibetica*

The results of the ADMIXTURE and SAMOVA analysis showed that the *L. tibetica* population was divided into two distinct groups (as illustrated in Fig. 2). Based on their geographical distribution, these groups were classified as Northern group and a Southern group (Fig. 1). The results of the ADMIXTURE and SAMOVA analysis confirmed the existence of two genetic groups: the Southern group in Tibet, China and the Northern group dispersed across Qinghai, Gansu and Sichuan provinces. Although most of the populations had a single genetic background, the populations (BS and JD) in the contact area between the two groups showed genetic introgression from the other group, as evidenced by the presence of a small proportion of genetic information from the other group. The BS population had a high proportion of genetic background from the Southern group, whereas the JD population was dominated by the Northern genetic background. This suggests that the two groups did not evolve in isolation from each other. The results of the PCA analysis (Fig. 3) supported the division of the *L. tibetica* populations into north and south groups. The Mantel test results (Additional file 4) confirmed a correlation between geographical distance and genetic structure in the *L. tibetica* populations. Our previous studies have established that *L. tibetica* is an ancient plant species of the QTP, which has undergone significant changes in terrain and climate over time [26]. During the Last Interglacial Maximum to the Last Glacial Maximum, the suitable distribution range of both the Northern and Southern populations of *L. tibetica* significantly decreased. Subsequently, during the mid-Holocene, their suitable habitats expanded considerably [26]. This is consistent with the historical population dynamics of *L. tibetica* predicted in this study. Both the Northern and Southern populations experienced a rapid decline in population size, followed by a gradual increase (Fig. 4).

The topography of the distribution area of *L. tibetica* populations in the QTP plays a crucial role in the differentiation of the Northern and Southern groups (Fig. 1). The major geographical barriers between these two groups include the Tanggula Mountains, the Nyainqentanglha Mountains, and the Salween River. The Northern group is predominantly influenced by the Asian monsoon and its habitats are characterized by a cold, dry climate and a relatively flat terrain [52]. On the other hand, the Southern group is predominantly influenced by the Indian monsoon, with a humid climate, higher precipitation levels, and a more complex topography consisting of mountains, valleys, and plateaus [52]. The Tanggula Mountains and the Nyainqentanglha Mountains have average elevations of 5000–6000 m, with the middle and high areas being covered in snow year-round. The area between the two mountains is considered an alpine meadow, with temperatures usually below the freezing point, particularly in winter, and low precipitation levels that primarily occur in the form of snow [53]. In addition, numerous studies also suggest that climate differences are one of the primary causes of genetic differentiation among species [54–56]. Therefore, we hypothesize that the insurmountable geographical barriers and resulting geographic isolation, coupled with climate differences, are the main factors contributing to the population structure of *L. tibetica* in the QTP.

The observed heterozygosity (*Ho*) and expected heterozygosity (*He*) are critical indicators of genetic diversity in plant populations as they provide insight into the evolutionary potential and adaptability of the population [57]. Statistical analysis revealed that the observed *Ho* values were significantly higher than the *He* values, indicating deviations from Hardy–Weinberg equilibrium. These deviations may be attributed to several factors, including recent genetic drift, which can prevent populations from reaching equilibrium, and gene flow between subpopulations that reduces homozygosity and increases heterozygosity [58]. Substructuring within populations or selection pressures acting on linked loci could further contribute to these patterns. Statistical analysis indicated that the observed *Ho* values for the BS (0.14995) and JD (0.14891) populations were significantly higher than the average *Ho* value of the other populations (0.13559, *p* < 0.001), confirming that these two populations exhibit higher genetic diversity. The higher genetic diversity could be explained by their geographic location at the interface of the Northern and Southern groups. This position likely facilitates gene flow and genetic admixture, contributing to their higher genetic diversity. Additionally, these populations may have maintained relatively stable effective population sizes and avoided significant demographic reductions, preserving genetic variation. In the study of *Primula tibetica*, *Potentilla glabra* and *Aconitum gymnandrum* populations in the same region, it has been observed that the populations distributed near the Hengduan Mountains undergo a process of separation, fusion, and re-separation over a prolonged period [59–61]. Although technical factors, such as sampling errors or genotyping inaccuracies, cannot be entirely ruled out, the consistent excess of heterozygosity observed across populations aligns with the influence of historical demographic fluctuations, geographic barriers, and climatic differences. These findings underscore the complex demographic and evolutionary processes that have shaped the genetic structure of *L. tibetica* populations in the QTP.

The observed heterozygote excess in *L. tibetica* populations, as indicated by negative *F*_*IS*_ values, likely arises from the interplay of historical demographic events and contemporary gene flow dynamics. Population bottlenecks during the Last Interglacial Maximum and Last Glacial Maximum substantially reduced genetic diversity through habitat contraction and amplified genetic drift [26]. Subsequent post-glacial expansion during the mid-Holocene enabled population recovery and genetic mixing through increased gene flow, effectively counteracting homozygosity accumulation and establishing the observed heterozygote surplus [62]. This phylogeographic structure aligns with patterns observed in other endemics of QTP and adjacent area. The topographical structure of the Mekong-Salween River region serves as a significant geographical barrier between the eastern and western populations of *Taxus wallichiana* and *Sinopodophyllum hexandrum*, resulting in genetic differentiation between the populations on either side of the mountains [63, 64]. The formation of the Nyainqentanglha Mountains has led to intraspecific differentiation in *Hippophae tibetana*, *Stuckenia filiformis*, and *Myriophyllum spicatum* [65–67]. However, geographical isolation is not always permanent. The *Juniperus tibetica* complex, *Osteomeles schwerinae*, and *Primula tibetica* groups have experienced partial population isolation followed by subsequent fusion [59, 68, 69]. These findings support our hypothesis that the BS and JD populations, located at the interface of the two genetic groups, may play a crucial role in shaping the genetic structure of *L. tibetica*. While these findings indicate that the BS and JD populations may have influenced the genetic structure of the Northern and Southern populations, caution should be exercised when drawing conclusions. The data are derived from neutral markers, which primarily reflect demographic history rather than adaptive processes.

The results of the AMOVA analysis indicated significant genetic variation between the Northern and Southern groups. This observation aligns with the findings reported by Tian et al. [70] and Chi et al. [71]. These results suggest that a significant geographical barrier exists between the Northern and Southern populations of *L. tibetica*. Although no morphological differences were observed between individuals in the Northern and Southern subgroups, genetic differences could accumulate over time due to evolutionary independence. This could lead to morphological differences, which could result in the formation of two or more distinct species that are unable to interbreed and produce viable offspring [72].

Previous studies utilizing chloroplast DNA, nuclear ribosomal DNA, and microsatellite markers established pronounced genetic divergence between northern and southern *L. tibetica* populations, attributing this pattern to isolation by the Tanggula-Nyainqentanglha mountain barrier and Pleistocene climatic fluctuations [26, 71]. While these markers identified major lineage splits corresponding to geographic boundaries, our genome-wide SNP analysis provides unprecedented resolution to dissect fine-scale genetic structure and historical demography. Consistent with Xia et al. [26], we confirm a deep genetic divide between northern and southern groups shaped by prolonged geographic isolation and climatic barriers. However, the enhanced resolution of SNPs, distributed uniformly across the genome, addresses key limitations of prior marker systems. This study reveals critical nuances: residual gene flow persists in contact zones, likely facilitated by glacial-interglacial range shifts that created episodic dispersal corridors; climatic oscillations drove cyclical population contractions and expansions, with contact zones potentially serving as microrefugia that preserved genetic diversity. High-resolution genomic data aids in revealing species-level evolutionary patterns and provides more precise insights into population historical processes [72]. Enhanced genomic data and more comprehensive species sampling have emerged as a new paradigm in systematic geography [73].

### Impact of global climate change and anthropogenic effects on *L. tibetica*

Species distribution models can provide valuable insight into the spatial patterns of species distribution. This is especially pertinent in regions characterized by complex topography and climate, such as the QTP [19, 74]. By projecting the current and future distribution potential of species, species distribution models can identify areas of crucial importance for conservation, informing the prioritization of protection and management efforts. Furthermore, these models can be used to evaluate the risk of extinction for individual species, guiding the development of targeted conservation strategies aimed at promoting species recovery [19, 75, 76].

Our integrative study reveals that distribution dynamics of *L. tibetica* on the QTP are shaped by elevation-driven climatic constraints and geographic isolation, consistent with its post-LGM diversification history [26]. Species distribution modeling identifies elevation as the primary niche determinant (Fig. 5), though temperature and precipitation synergistically regulate habitat suitability. By 2100, these combined pressures are projected to drive upward range shifts, disproportionately threatening southeastern plateau populations (e.g., BS, SD, HZ) through habitat contraction. Conversely, central Tibet and southeastern Qinghai (including DX, LZ, MLS, JD, ZQ, YS, XLX, ZD, QML, QL, MY, XHZ, GD, TJ and GH populations) retain climatic suitability, warranting designation as in situ conservation priorities. This conservation imperative is amplified by the species’ dual vulnerability: as a culturally significant Tibetan medicinal resource [26], *L. tibetica* faces unsustainable harvesting due to limited cultivation practices [71]. Field surveys confirm severe wild population declines, necessitating ex situ protection for genetically diverse populations (e.g., BS, JD) exhibiting high heterozygosity and unique alleles [76]. Therefore, combining the results of the species distribution models result, it is recommended that in situ conservation efforts be focused on the Lhasa region and surrounding areas, with the BS and JD populations prioritized for commercial cultivation development.

## Conclusion

In this study, we utilized population genetics to gain a deeper understanding of the population genetic structure of the endemic species *L. tibetica*, in the QTP. SNP-based analyses provided finer-scale resolution, allowing us to capture subtle variations in genetic diversity and gene flow across the plateau. Our analyses confirmed that the distribution of *L. tibetica* on the QTP is separated into Northern and Southern parts by the Tanggula and Nyainqentanglha Mountains, leading to the possibility of primary stage of species differentiation due to geographic isolation and significant climatic differences. Demographic modeling indicated significant population dynamics of *L. tibetica*, including a bottleneck event around 0.32 Ma, continuous gene flow between Northern and Southern groups, and a recent expansion of effective population size. Species distribution models reveal that elevation is the most significant factor influencing the distribution of *L. tibetica*, followed by precipitation and temperature. Under future global warming scenarios, suitable habitats for *L. tibetica* populations are expected to decline significantly, while the southeastern edge of the QTP faces an increased risk of extinction. Considering the identified threats and shifting ecological dynamics, we recommend concentrating in situ conservation initiatives in the Lhasa region and surrounding areas, preserving the genetic diversity of local populations. Meanwhile, to mitigate the adverse effects of over-harvesting and ensure the sustainable supply of *L. tibetica*, our findings underscore the necessity to prioritize populations with high heterozygosity and unique genetic components for commercial cultivation.

## Supplementary Information

Additional file 1.

Additional file 2.

Additional file 3.

Additional file 4.

Additional file 5.

Additional file 6.

Additional file 7.

Additional file 8.

Additional file 9.

## Data Availability

The datasets generated or analyzed during this study are included in this article (and its Additional file) or are available from the corresponding author on reasonable request. All raw data were submitted to the NCBI Sequence Read Archive: BioProject ID PRJNA1140884.

## Abbreviations

QTP: Qinghai-Tibet Plateau
kya: Kilo years ago
Ma: Million years ago
NGS: Next generation sequencing
SNPs: Single nucleotide polymorphisms
CV error: Cross-validation error
GBS: Genotyping-by-sequencing
CTAB: Cetyltrimethylammonium bromide
2D-SFS: Joint folded site frequency spectrum
HWE: Hardy–Weinberg equilibrium
AIC: Akaike's Information Criterion
SDM: Species distribution modeling
DEM: Digital Elevation Model
AUC: Area under the curve
ROC: Receiver operating characteristic
