# Supplementary material for: Genetic structure and conservation implications of Lancea tibetica (Mazaceae), a traditional Tibetan medicinal plant endemic to the Qinghai- Tibet Plateau
Source: BMC Plant Biol. 2025 Feb 18;25:222. doi: 10.1186/s12870-025-06258-7 (PMC11834613; doi:10.1186/s12870-025-06258-7)
Supplement: Supplementary file 3 — Additional file 3. [file 12870_2025_6258_MOESM3_ESM.pdf]

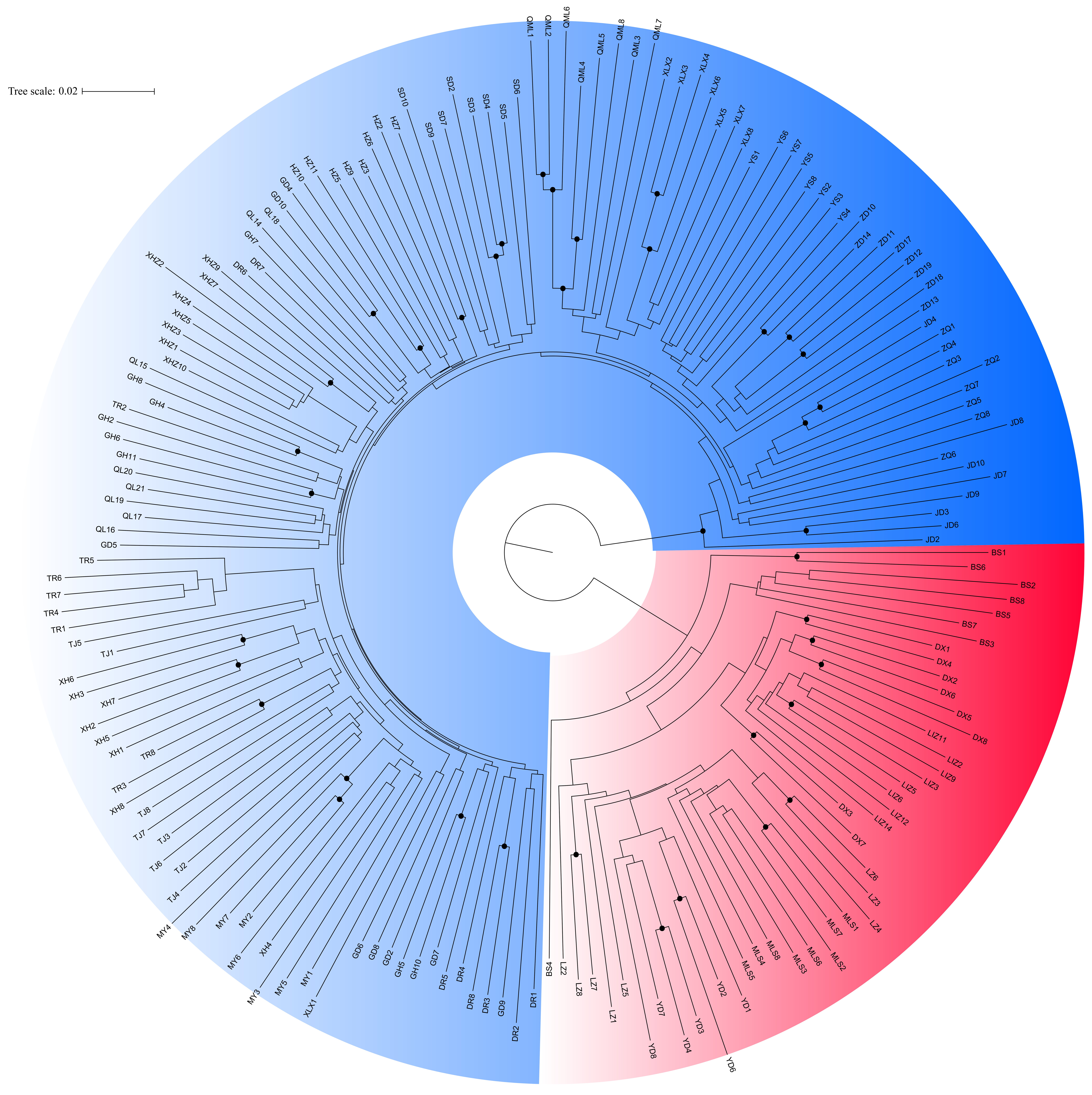

Additional file 3 The neighbor-joining phylogenetic tree of 183 Lancea tibetica individuals. Branches with bootstrap>70% are marked with black dots. The branches with red background represent the Southern group, while those with blue background represent the Northern group.
