# Supplementary material for: Genetic structure and conservation implications of Lancea tibetica (Mazaceae), a traditional Tibetan medicinal plant endemic to the Qinghai- Tibet Plateau
Source: BMC Plant Biol. 2025 Feb 18;25:222. doi: 10.1186/s12870-025-06258-7 (PMC11834613; doi:10.1186/s12870-025-06258-7)
Supplement: Supplementary file 7 — Additional file 7. [file 12870_2025_6258_MOESM7_ESM.pdf]

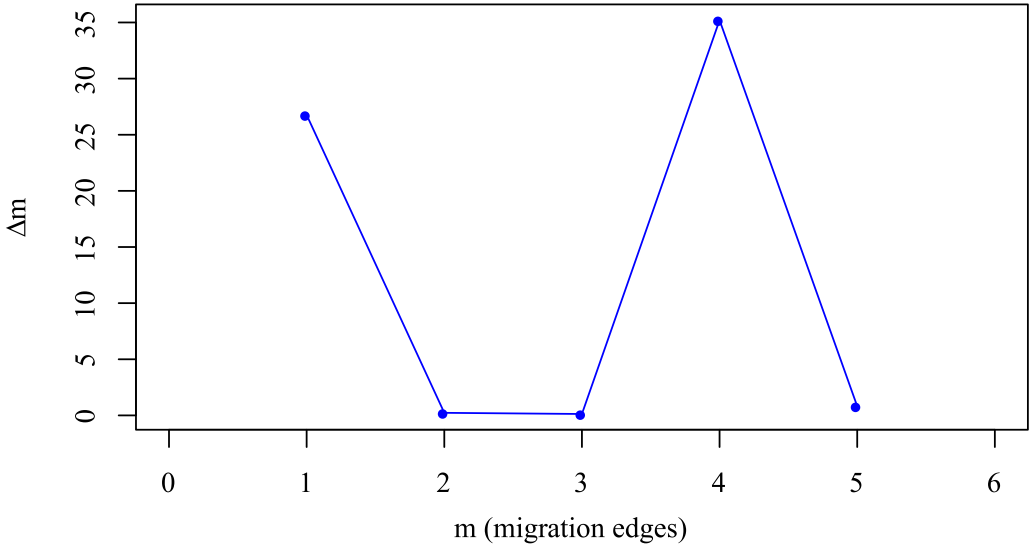

Additional file 7 Simulate the optimal migration edges of the northern group of *Lancea tibetica* populations.
