# Supplementary figures and images for: Genetic structure and conservation implications of Lancea tibetica (Mazaceae), a traditional Tibetan medicinal plant endemic to the Qinghai- Tibet Plateau
Source: BMC Plant Biol. 2025 Feb 18;25:222. doi: 10.1186/s12870-025-06258-7 (PMC11834613; doi:10.1186/s12870-025-06258-7)

Average Sensitivity vs. 1 - Specificity for lancea

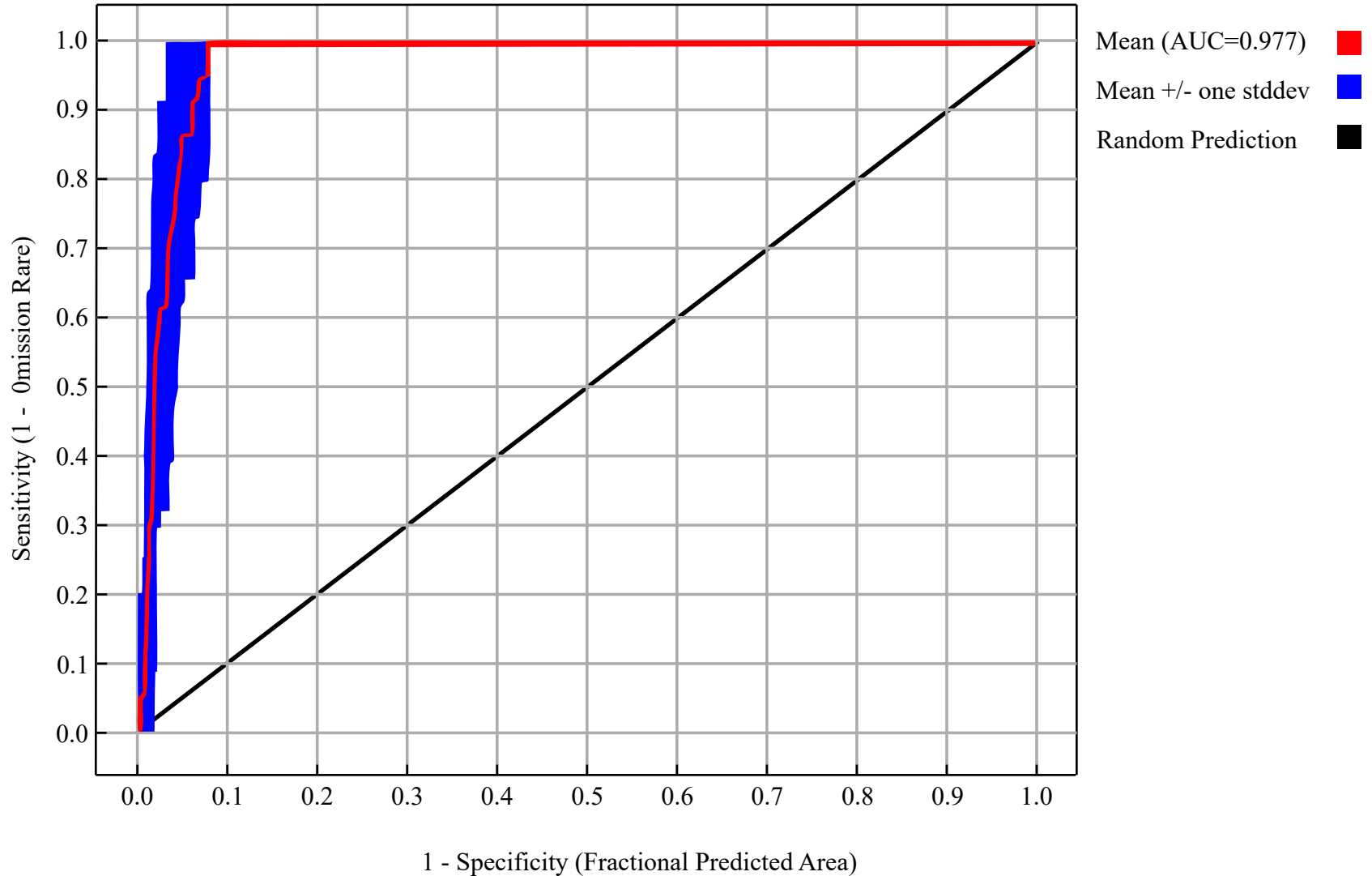

Supplement: Supplementary file 9 — Additional file 9. [file 12870_2025_6258_MOESM9_ESM.pdf]
